# *Vipera aspis* Envenomation in a Dog from Central Italy: Clinical Observations and Therapeutic Considerations

**DOI:** 10.3390/vetsci13010049

**Published:** 2026-01-05

**Authors:** Giulio Mannocchi, Filippo Roberto Busardò, Luigi Tonino Marsella, Roberta Tittarelli

**Affiliations:** 1 Department of Biomedicine and Prevention, Section of Legal Medicine, Social Security and Forensic Toxicology, Faculty of Medicine and Surgery, University of Rome “Tor Vergata”, Via Montpellier 1, 00133 Rome, Italy; marsella@uniroma2.it (L.T.M.); roberta.tittarelli@uniroma2.it (R.T.); 2 Veterinary Diagnostics North Rome, Via Roccaraso 7/c, 00135 Rome, Italy; filippo.busardo@hotmail.it

**Keywords:** envenomation, dog, *Vipera aspis*, clinical course, hematological findings, supportive therapy, toxicology

## Abstract

*Vipera aspis* envenomation represents a potentially life-threatening condition in dogs, particularly in rural or semi-rural areas of Central Italy, where this species is endemic. This case report describes the clinical presentation, diagnostic findings, therapeutic management, and outcome of a dog bitten by *Vipera aspis* in the Umbria region. At admission, the dog exhibited mild fever, localized swelling, pain at the bite site, and mild systemic clinical signs. Antivenom was not administered, and the dog was managed with supportive and medical therapy aimed at controlling inflammation, preventing secondary infection, and monitoring systemic involvement. The clinical course was favorable, and the dog recovered without complications. As this report describes a single clinical case, no conclusions regarding treatment efficacy can be drawn. However, this case provides additional clinical information on the management and outcome of *Vipera aspis* envenomation in dogs.

## Simple Summary

This report describes the clinical course of a dog bitten by a viper in Umbria, a region of central Italy where *Vipera aspis* is commonly found. Viper envenomation can represent a serious clinical condition in dogs and typically requires prompt veterinary evaluation. The dog presented with mild fever, local swelling, pain at the bite site, and systemic clinical signs. Antivenom was not administered, and the dog was managed with supportive and medical therapy aimed at controlling inflammation, preventing secondary infection, and monitoring systemic involvement. The clinical evolution was favorable, and the dog recovered without any complication. As this report describes a single clinical case, no conclusions regarding treatment efficacy can be drawn. However, this case contributes additional clinical information on the management and outcome of *Vipera aspis* envenomation in dogs and highlights the need for further studies to better characterize optimal treatment strategies.

## 1. Introduction

Snakebite envenomation remains a relevant medical concern worldwide, with significant morbidity and mortality reported each year [1]. In Europe, venomous snakebites are primarily caused by species of the family Viperidae, genus Vipera, which includes the only native venomous snakes on the continent. Among the fourteen species of “true vipers” (Viperinae) present in Europe, six are considered of major medical importance: *Vipera ammodytes*, *Vipera aspis*, *Vipera berus*, *Vipera latastei*, *Vipera seoanei*, and *Vipera ursinii* [2]. In Italy, snakebite cases are not subject to mandatory reporting, contributing to an underestimation of their true incidence and clinical impact [3,4]. The most commonly encountered venomous snake is the Asp viper (*Vipera aspis*), whose venom contains at least 64 identified proteins, including hemotoxins and phospholipases A2 as predominant components [5]. Viper venom is a complex mixture of bioactive molecules, such as proteases, serine proteases, metalloproteinases, phospholipases A2, and prothrombin activators, which may induce local tissue injury, systemic inflammation, coagulopathy, and, in severe cases, multiorgan involvement (Table 1) [2,6,7].

Although Viperidae venom is typically characterized by hemotoxic and cytotoxic effects, neurological complications have also been reported in humans in Italy and Switzerland [15,18]. In dogs, viper envenomation is commonly associated with local swelling, edema, pain, and hematoma at the bite site, while systemic clinical signs may include anemia, thrombocytopenia, coagulopathy, hemolysis, and, less frequently, renal involvement [19,20]. Gastrointestinal signs are uncommon in dogs compared to humans.

Dogs are particularly exposed to snakebites due to their inquisitive behavior and frequent outdoor activity, especially in rural and mountainous areas during warmer months when vipers are most active. Despite this, published data on *Vipera aspis* envenomation in dogs remain limited. While a prospective multicenter study has evaluated antivenom administration in dogs bitten by *Vipera* species, available reports mainly involve medium-sized hunting breeds [21]. To date, no detailed case reports describing *Vipera aspis* envenomation in small-sized dogs in Italy have been published.

The present case report describes the clinical presentation, diagnostic findings, therapeutic management, and outcome of a small-breed dog bitten by *Vipera aspis* in central Italy.

## 2. Case History

A four-year-old, 4.5 kg, spayed female mixed-breed dog was examined in September, approximately 40 min after a suspected viper bite. The envenomation event was not directly observed; however, the owners reported seeing a viper moving away from the dog, and the snake was identified as *Vipera aspis* based on the geographical area and description provided.

On admission, vital parameters were as follows: body temperature 39.4 °C (reference range [rr], 37.8–39.2 °C), heart rate 130 beats per minute (rr, 60–140 bpm), and respiratory rate 20 breaths per minute (rr, 10–35 breaths per minute). Breathing was eupneic, although the dog was panting. The dog was unable to stand but remained mentally alert.

Mucous membranes were pink and mildly congested. Cardiopulmonary auscultation was unremarkable, and abdominal palpation revealed a soft, non-painful abdomen. Localized swelling with skin ecchymosis was observed on the left hind limb at the level of the tarsus. The affected area was warm and mildly painful on palpation, consistent with a local inflammatory response to viper envenomation.

Initial hematological evaluation revealed a packed cell volume (PCV) of 50.9% (rr, 37.3–61.7%) and total solids (TS) of 5.8 g/dL (rr, 5.2–8.2 g/dL). Coagulation tests were not performed based on the clinical judgment of the attending veterinarians, given the mild and localized clinical presentation and the absence of overt signs of systemic coagulopathy at admission.

Early treatment included intravenous dexamethasone (Dexadreson^®^, MSD Animal Health S.r.l., Segrate, Milan, Italy) administered at 0.25 mg/kg to modulate inflammation and reduce the risk of edema. Antibiotic therapy with amoxicillin/clavulanic acid (Synulox^®^, Zoetis Italia S.r.l., Rome, Italy) was given at a dosage of 20 mg/kg every 12 h. Supportive therapy also included administration of a mefepronic acid-based hepatoprotective supplement (Hepagen^®^, Fatro Industria Farmaceutica Veterinaria S.p.A., Ozzano dell’Emilia (BO), Bologna, Italy) due to mild increases in liver enzyme activity (alanine aminotransferase [ALT]: 137 U/L; aspartate aminotransferase [AST]: 210 U/L; alkaline phosphatase [ALP]: <10 U/L; gamma-glutamyltransferase [GGT]: 0 U/L), as a precautionary measure to support hepatic function [22].

**Figure 1:**
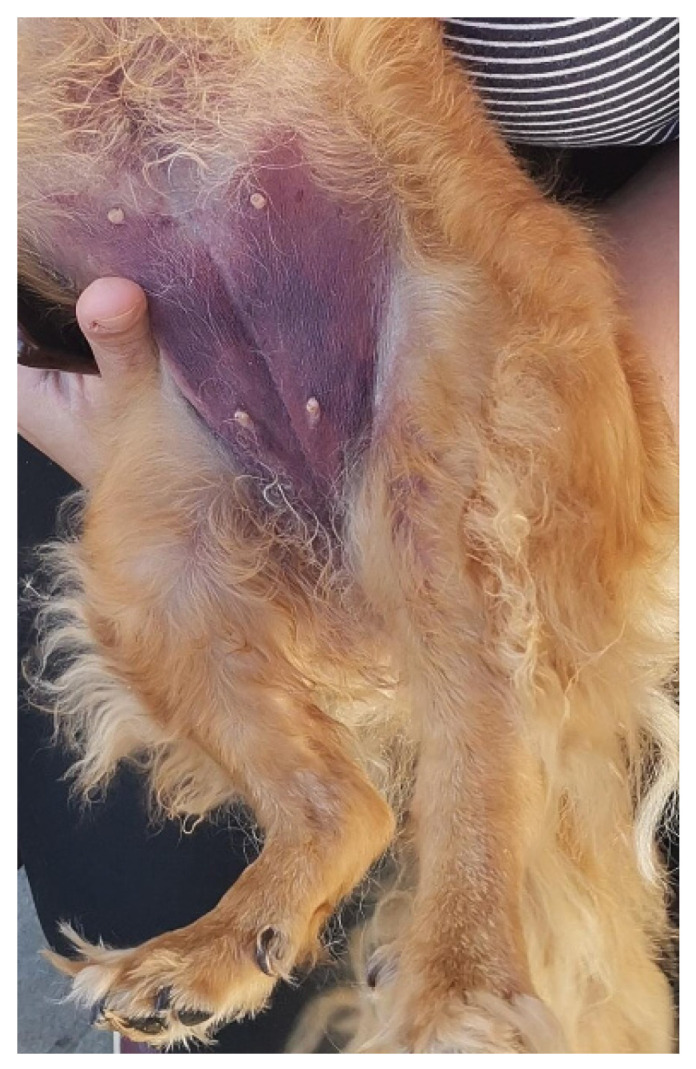
Skin ecchymosis involving the ventral abdominal region, observed within the first 24 h after admission.

During hospitalization, the skin ecchymosis and swelling extended from the tarsal region to involve the ventral abdominal area (Figure 1). The affected limb and abdominal region were treated daily with a topical gel containing escin and phosphatidylcholine (Essaven Gel^®^, Cooper Consumer Health IT S.r.l., Paris, France), compounds with anti-oedematous and venotonic properties [23].

Over the following days of hospitalization, the dog’s clinical condition progressively improved, with return of voluntary feeding, gradual resolution of the skin ecchymosis infiltration, and no requirement for blood transfusion. The dog was discharged on the fifth day of hospitalization and continued to recover without complications.

Post-discharge therapy included amoxicillin/clavulanic acid (Synulox^®^, Zoetis Italia S.r.l., Rome, Italy) for 7 days, a liver support supplement (Besame^®^, Candioli Pharma, Beinasco (TO), Turin, Italy) for 30 days, and a palatable escin-based paste (Escinapet^®^, AVICENNA, Avellino, Italy) for 10 days to support circulation and microcirculation.

Hematochemical analyses were performed daily during hospitalization, with follow-up evaluations at 15 and 50 days after discharge to monitor hematological and clinical evolution (Table 2).

## 3. Discussion

Viper envenomation in dogs represents a medical emergency with highly variable clinical presentations, influenced by several factors including the snake species involved, the amount of venom injected, the anatomical location of the bite, and the time elapsed before veterinary intervention. Accurate diagnosis may be challenging, particularly when the envenomation event is not directly observed or when the snake species cannot be reliably identified, potentially delaying appropriate management.

This case report describes the clinical course of a small-breed dog bitten on the left hind limb by a viper presumed to belong to the *Vipera aspis* species, based on geographic distribution and morphological description. The dog developed rapid-onset local inflammation characterized by swelling, pain, and mild hyperthermia, findings commonly reported in viperid envenomation. Hematological abnormalities observed during hospitalization, including regenerative anemia, leukocytosis, mild thrombocytopenia, and monocytosis, are consistent with previously described systemic responses to viper venom and reflect inflammation, vascular injury, and hematologic consumption processes.

Viper venom contains multiple bioactive components, including metalloproteinases and procoagulant enzymes, which are known to induce endothelial damage, increased capillary permeability, and disturbances in hemostasis. These mechanisms may account for the progressive extension of edema and skin ecchymosis observed in this case, as well as the hematologic changes documented during hospitalization. However, the absence of coagulation testing in this patient precludes definitive conclusions regarding the presence or extent of systemic coagulopathy.

The severity of envenomation is known to depend on several variables, including the quantity of venom injected, which itself may be influenced by the snake’s size, age, and physiological state, as well as environmental factors such as seasonality. Although early veterinary presentation and seasonal factors may have influenced the clinical course in this case, their specific impact cannot be determined with certainty.

Currently, no standardized treatment protocol exists for canine viper envenomation, and therapeutic approaches reported in the literature vary widely. Antivenom administration is generally considered the definitive treatment in cases of moderate to severe envenomation; however, its use may be limited by availability, cost, or clinical judgment. In this case, antivenom was not administered, and the dog was managed with supportive and medical therapy, including anti-inflammatory and antimicrobial agents, alongside close clinical and laboratory monitoring.

The use of corticosteroids and antibiotics in snakebite management remains controversial in both human and veterinary medicine. While corticosteroids are not routinely recommended in human envenomation due to insufficient evidence of benefit, they are sometimes employed in veterinary practice for their anti-inflammatory properties. In the present case, dexamethasone may have contributed to the control of local inflammation; however, its role cannot be conclusively established. Similarly, although secondary infections following viper bites are uncommon, antimicrobial therapy may be considered in selected cases due to the potential introduction of oral flora or opportunistic pathogens at the bite site.

The favorable clinical outcome observed in this dog, characterized by gradual resolution of clinical signs and normalization of hematological parameters over time, reflects the natural clinical course following management of envenomation in this individual case. Importantly, this case report does not provide direct evidence regarding the efficacy of any specific treatment modality, nor does it allow conclusions to be drawn regarding the necessity or omission of antivenom therapy.

In conclusion, this case contributes additional clinical information on *Vipera aspis* envenomation in a small-breed dog and highlights the importance of individualized clinical assessment, supportive care, and careful monitoring. Further prospective studies are required to better define optimal management strategies and to clarify the role of non-antivenom therapies in canine viper envenomation.

## 4. Conclusions

This case report describes the clinical course and management of a dog with presumed *Vipera aspis* envenomation that was treated without antivenom administration. Although the favorable outcome observed in this case suggests that supportive and medical management may be sufficient in selected cases, no conclusions can be drawn regarding the efficacy of any specific therapeutic approach based on a single clinical observation.

Timely veterinary evaluation, careful clinical monitoring, and individualized supportive care remain essential components in the management of canine viper envenomation. This report contributes additional clinical information to the limited veterinary literature on *Vipera aspis* bites in dogs, particularly in small-breed patients, and highlights the need for further controlled studies to better define optimal treatment strategies and the role of antivenom therapy in different clinical scenarios.

## Figures and Tables

**Table 1:** Major constituents of viper venom.

Enzyme	Effects	Reference
Proteases	Destroy the endothelium and basal membrane of capillaries causing soft tissue damage and necrosis	[8,9]
Serine proteases	Pro-coagulant activity, conversion of fibrinogen in fibrin, platelet aggregation, activators of plasminogen, factor X, factor V, prothrombin and protein C.	[10]
Metalloproteinases	Involved in systemic inflammation, fibrinolytic activity, necrosis at the site of envenomation	[11]
Metalloproteinases hemorrhagin 1 and 2	Disrupt platelet function, prolong both prothrombin time (PT) and partial thromboplastin time (PTT)	[9]
Phospholipase A2	Causes red blood cell abnormalities (echinocytes, spherocytes), platelet aggregation, thrombocytopenia and muscle damage	[12]
Prothrombin activators	Induce intravascular coagulopathy (DIC) and defibrination	[13]
Hyaluronidase	Decreases the strength in connective tissue allowing the venom to spread more easily throughout the body	[14]
α-Neurotoxins	Exhibit postsynaptic activity and may interfere with the functioning of voltage-dependent ion channels	[15,16]
β-Neurotoxins	Act at presynaptic site inhibiting the release of the acetylcholine	[16,17]

**Table 2:** Chronological evolution of hematological values and clinical signs.

Day	PCV (%)	Platelets (×10^3^/µL)	Clinical Notes
0	50.9	180	Arrival; local edema; pain
1	37.1	140	Escin + fluids
2	31.7	110	Hemorrhagic infiltration
3	36.0	135	Improvement
4	37.6	150	Edema reduced
15	42.5	220	Full recovery
50	47.8	260	Stable

## Data Availability

The original contributions presented in this study are included in the article. Further inquiries can be directed to the corresponding author.
